# Pharmacokinetic interactions and clinical implications of PPIs and CDKIs in breast cancer: a systematic review and meta-analysis

**DOI:** 10.1186/s13643-025-03046-0

**Published:** 2025-12-30

**Authors:** Agnese Graziosi, Roberto Pane, Emanuele Tinazzo, Marco Basso, Matteo Avantaggiato, Alice Schianchi, Mattia Canella, Mauro Melis, Alessandro Nani, Marzia Del Re, Romano Danesi, Arianna Pani, Riccardo Giossi, Diego Fornasari

**Affiliations:** 1 Department of Medical Biotechnology and Translational Medicine, Postgraduate School of Clinical Pharmacology and Toxicology, Università Degli Studi Di Milano, Milan, Italy; 2 SC Territorial Pharmacy, Pharmaceutical Department, ASL Alessandria, Alessandria, Italy; 3 Division of Clinical Pharmacology and Toxicology, Institute of Pharmacological Sciences of Southern Switzerland, Ente Ospedaliero Cantonale, Lugano, Switzerland; 4 Saint Camillus International University of Medical and Health Sciences, Rome, Italy; 5 Department of Oncology and Hemato-Oncology, University of Milan, Milan, Italy; 6 Poison Control Center and Clinical Pharmacology Unit, ASST Grande Ospedale Metropolitano Niguarda, Milan, Italy

**Keywords:** Proton pump inhibitors, Cyclin-dependent kinase 4/6 inhibitor, Breast Cancer, Drugs Interaction

## Abstract

**Background:**

Breast cancer is the fourth leading cause of cancer mortality worldwide. New drugs, such as cyclin-dependent kinase 4/6 inhibitors (CDKIs), increase the life expectancy of receptor-positive (HR+) and human epidermal growth factor receptor 2 negative (HER2-) breast cancer patients. This class acts to limit the G1/S transition in tumor cells, inducing tumor cell death. Owing to the basic nature of CDKIs, their solubilities are pH dependent and could be influenced by the concurrent use of acid-reducing agents such as proton pump inhibitors (PPIs). This meta-analysis aims to assess the impact of co-administering PPIs on the pharmacokinetics and clinical efficacy of CDKIs in breast cancer patients.

**Methods:**

Four databases with English-language restriction were searched for concomitant CDKIs and PPIs keywords from their inception date to 2024 March 7th. Prospective, retrospective, randomized or nonrandomized clinical studies and observational longitudinal studies with at least one outcome of interest were included. The outcomes included pharmacokinetic variables, progression-free survival (PFS), and overall survival (OS).

**Results:**

We included three pharmacokinetic studies conducted in patients enrolled in clinical trials and seven clinical studies evaluating survival outcomes. When coadministered with palbociclib, PPIs significantly reduced the serum maximum concentration (Cmax) (MD -35.37 ng/mL; 95%CI from -67.59 to -3.16) and increased the clearance (CL/F) (MD 61.24 L/h; 95%CI from 14.66 to 107.82). Ribociclib Cmax and AUC did not significantly differ among the PPIs users. However, the overall PFS favored PPIs non-users (HR 1.74; 95%CI from 1.28 to 2.37). Consistently, coadministration of PPIs with CDKIs significantly increased the likelihood of reduced OS (HR 1.99; 95%CI from 1.18 to 3.33). The effect was confirmed only for the palbociclib subgroup (HR 2.11; 95%CI from 1.17 to 3.81). No data were available for OS evaluation with ribociclib. A single study on abemaciclib revealed nonsignificant differences (HR 1.30; 95%CI from 0.53 to 3.19), with similar results for OS.

**Conclusions:**

PPI use in HR + /HER2- breast cancer patients treated with palociclib should be avoided. When strictly necessary, ribociclib may be preferred to palbociclib, even though closer monitoring is strongly advised.

**Systematic review registration:**

PROSPERO identifier number CRD42024506456.

**Supplementary Information:**

The online version contains supplementary material available at 10.1186/s13643-025-03046-0.

## Background

In 2022, breast cancer accounted for nearly 2.3 million new cases in women globally and was the fourth most common cause of cancer-related mortality [1]. Approximately 70% of cases are hormone receptor-positive (HR +) and human epidermal growth factor receptor 2 negative (HER2 −) [2]. Almost a decade ago, the approval of the first cyclin-dependent kinase 4/6 inhibitor (CDKI) established a milestone in the treatment of HR +/HER2- advanced breast cancer [3]. The mechanism of action of CDKIs is to prevent retinoblastoma protein (Rb) phosphorylation and the consequent activation of E2F transcription factor through their binding to the CDK4/6-Cyclin D-p21/p27 complex, thus preventing progression from the G1 phase to the S phase and inducing a senescence-like status in tumor cells. Other indirect mechanisms of action have been proposed and are discussed elsewhere [4, 5]. Currently, three CDKIs are available in clinical practice: palbociclib, ribociclib, and abemaciclib. Despite some chemical and pharmacological differences, they have shown remarkable benefits in terms of progression-free survival (PFS) and overall survival (OS) in combination with aromatase inhibitors or fulvestrant [6–20]. Due to the basic nature of CDKIs, their solubility is pH-dependent, thus, their absorption could be influenced by the concurrent use of acid-reducing agents, such as proton pump inhibitors (PPIs) [21–24]. PPIs are widely used drugs and influence the bioavailability of several anticancer drugs, such as tyrosine kinase inhibitors and immune checkpoint inhibitors, by increasing the gastric pH [23, 25]. Some studies have investigated whether the use of PPIs could influence the clinical outcomes of patients taking CDKIs [26–33]. However, the results revealed discrepancies in terms of PFS and/or OS. For this reason, we conducted a systematic review and meta-analysis to evaluate the effects of PPIs on the bioavailability and pharmacokinetics (PK) of CDKIs and the resulting clinical outcomes.

## Methods

### Search strategy and study selection

We searched PubMed, Embase, the Cochrane Library, and Web of Science from their inception date to 7th March 2024, with English-language restrictions, to retrieve eligible studies. The search strategy included “palbociclib”, “ribociclib”, “abemaciclib”, and “PPI” as keywords, both as MeSH and free terms with related synonyms, combined with Boolean operators, as applicable. The complete search strategy is available in Supplementary Table S2. Three investigators (A.G., R.P. and E.T.) independently screened the retrieved records by title and abstract. All potentially relevant articles were read in full for a final decision on inclusion. Any discrepancies were collegially discussed and resolved. Eligibility criteria were defined according to the PICO framework. The population included patients with breast cancer treated with CDKIs. The intervention was the concomitant use of PPI. The comparator was CDKIs treatment without PPIs. Outcomes of interest included pharmacokinetic parameters (Cmax, AUC, CL/F) and survival outcomes, either PFS or OS. No restrictions were applied regarding the specific quantitative study design; thus, randomized controlled trials, nonrandomized clinical trials, and prospective or retrospective observational studies were eligible if they reported at least one outcome of interest.. In addition, the reference lists of relevant systematic reviews and meta-analyses were manually screened to identify any additional eligible studies not captured by the electronic search. Non-English-language studies, conference abstracts, noncomparative case series, case reports, systematic reviews, and meta-analyses were excluded. Our study was registered on PROSPERO (CRD42024506456), and we followed the Preferred Reporting Items for Systematic Reviews and Meta-analysis (PRISMA) statement for the realization of this work as shown in the PRISMA checklist (Supplementary Table S1) [34].

### Data extraction and assessed outcomes

Data concerning the authors, study year, study design, number of participants and relative repartition in study arms, including CDKIs and PPIs and the relative number of participants exposed, sex, mean/median study age, Eastern Cooperative Oncology Group (ECOG) performance score, endocrine sensitivity, menopausal status, presence of metastatic disease, previous treatment lines, Ki-67 status, feeding state at the moment of the CDKI assumption, and clinical outcomes were extracted. The PK outcomes included the serum maximum concentration (Cmax) expressed in ng/mL, the area under curve (AUC) expressed in mg·h/mL, and the apparent oral clearance (CL/F) expressed in L/h. PK outcomes have been extracted as arithmetic or geometric means with associated standard deviation (SD) or percentual coefficient of variation (CV%). Clinical outcomes included PFS and OS expressed as hazard ratios (HR) with associated 95% confidence interval (95%CI). When both adjusted and unadjusted results were provided in the included studies, we extracted adjusted results to be included in our meta-analysis.

### Risk of bias assessment

The Cochrane Risk of Bias in Non-randomized Studies of Interventions (ROBINS-I) tool was used to independently assess the risk of bias of each article that satisfied the eligibility criteria by two pairs of authors (M.A and M.C.; A.G. and R.P.). The tool is divided into 7 bias domains: confounding, selection of participants in the study, classification of interventions, deviations from intended interventions, missing data, measurement of outcomes, and selection of the reported result. For the ROBINS-I tool, the prespecified confounding domains we considered relevant to address in the in included studies were age, sex, menopausal status, ECOG score, previous lines of treatment, presence of metastasis, sites of metastasis, and Ki-67. We also checked for the fed state at the moment of the treatment assumption; however, we did not consider it necessary to control for this latter variable. Since we wanted to investigate the possible interaction effect of PPIs on the efficacy and pharmacokinetics of CDKIs, we decided that our study aimed to verify the adhering to the intervention [35]. Controversies were resolved through discussion among all the authors. The risk of bias figures was realized with the online robvis tool.

### Statistical analysis

When at least 2 studies had available data, we performed a random-effects generic inverse variance to estimate pooled HR and mean differences (MD) with associated 95%CI. We assessed heterogeneity with I^2^ statistics. Publication bias was assessed with funnel plots. Cochrane RevMan 5.4 software was used for all analyses [36].

### Summary of findings

We used GRADEproGDT to produce a summary of findings for our meta-analysis. The quality of evidence was independently evaluated with the GRADE method by two authors (R.P. and R.G.), and discrepancies were resolved by discussion.

## Results

### Study characteristics

**Fig. 1:**
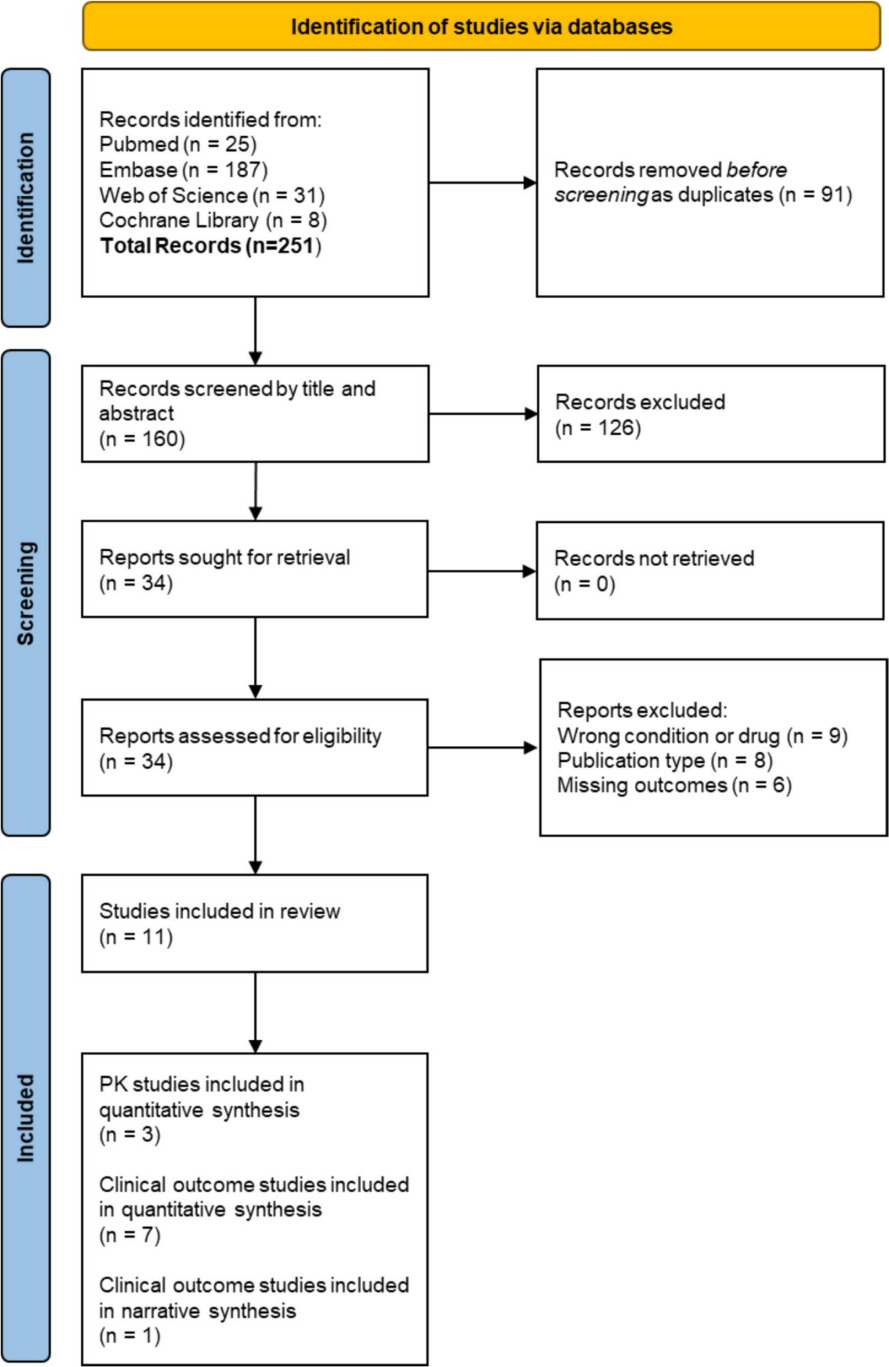
PRISMA flow chart

The systematic literature search identified a total of 251 articles (Fig. 1). After checking for duplicates, 91 were removed. At the end of the screening process, 34 articles were read in full. Ten studies met the eligibility criteria and were ultimately included in the meta-analysis [26–28, 30–33, 37–40]. One study reported our outcomes of interest but did not provide usable results for quantitative analysis; hence it was included in the systematic review and narratively discussed [29].

Among those, three evaluated the effect of PPI coadministration on the PK of CDKIs in a total of 173 participants (81 receiving palbociclib and 92 ribociclib). Among them, 73 received concomitant PPIs (42.4%). The enrolled participants were either all male, close to equity, all female, or not reported. Race was not reported in the majority of the included articles. Only one study specified races with a higher prevalence of white participants [37]. One study developed a highly predictive PK model for CDKIs starting from randomized open-label study samples (NCT04484064) [40]. Another study evaluated the effects of PPIs on the PK of CDKIs in patients from three different clinical trials, a randomized parallel trial (NCT01872260) and two nonrandomized single-group assignment trials (NCT01898845 and NCT01237236) [38]. A third study reported the results of two other phase I, open-label, crossover trials (NCT02097329, NCT01918176) [37]. The characteristics of the included PK outcome studies are detailed in Table 1 and Supplementary Figure S1.

**Table 1:** PK outcome studies characteristics and results

Study, year	Study design	Sex (%)	Age, mean (SD)	Race (n)	Treatment arm (n)	Reported PPI	Cmax,Mean (SD)	Cmax, gMean (CV%)	AUC, gMean (SD)	AUC, gMean (CV%)	CL/F, Mean (SD)
Courlet, 2022 [40]	NRCT	F (100)	65 (4.6)	NA	Palbociclib (33)	\	NA	NA	NA	NA	67 (3.35)
					Palbociclib + PPI (11)	NA	NA	NA	NA	NA	131 (5.24)
Samant, 2018a [21, 38]	NRCT	NA	NA	NA	Ribociclib (10/13)^a^	\	NA	1.62 × 10^3^ (53.2)	NA	21.1 (57.2)	NA
					Ribociclib + PPI (8/10)^a^	NA	NA	1.78 × 10^3^ (34.6)	NA	24.7 (30.6)	NA
Samant, 2018b [21, 38]	NRCT	NA	NA	NA	Ribociclib (6/6)^a^	\	NA	3.5 × 10^3^ (65.8)	NA	55.1 (68.6)	NA
					Ribociclib + PPI (2/2)^a^	NA	NA	2.7 × 10^3^ (53.0)	NA	42.6 (28.7)	NA
Samant, 2018c [21, 38]	NRCT	NA	NA	NA	Ribociclib (46/48)^a^	\	NA	1.87 × 10^3^ (60.3)	NA	23.7 (61.3)	NA
					Ribociclib + PPI (12/13)^a^	Rabeprazole	NA	2.05 × 10^3^ (74.7)	NA	25.9 (79.1)	NA
Sun, 2017a [22]	OLCO	F (42.3)	45.4 (8.1)	W (25), B (1), O (0)	Palbociclib (22)	\	65.28 (21.50)	61.74 (36)	1.971 (0.55)	1.90 (38)	66.78 (20.37)
		M (57.7)			Palbociclib + PPI (23)	Rabeprazole	13.37 (6.14)	12.25 (44)	0.723 (0.29)	0.673 (40)	176.2 (61.81)
Sun, 2017b [37]	OLCO	M (100)	34.9 (7.4)	W (3), B (15), O (9)	Palbociclib (14)	\	51.76 (7.84)	51.21 (15)	1.551 (0.30)	1.524 (20)	80.78 (16.04)
					Palbociclib + PPI (14)	NA	32.72 (12.78)	30.30 (44)	1.352 (0.42)	1.302 (28)	94.51 (23.15)

*Abbreviations: AUC* area under curve, *B* black, *CL/F* apparent oral clearance, *Cmax* serum maximum concentration, *CV%* percentual coefficient of variation, *F* female, *gMean* geometric mean, *M* male, *NA* not available, *NRCT* non-randomized clinical trial, *O* other, *OLCO* open-label crossover trial, *PPI* proton pump inhibitor, *W* white, *SD* standard deviation

Cmax, AUC, and CL/F values are reported as ng/mL, mgh/mL, and L/h, respectively. When not reported as gMean, Cmax and AUC values are given as arithmetic mean and SD

^a^The first number in brackets refers to the sample size reported for AUC measurements while the second number refers to the sample size reported for Cmax measurements

The remaining 7 articles included in the meta-analysis, delved into the effects of concomitant PPI administration on CDKI survival outcomes (i.e., PFS and/or OS). All of them were retrospective observational studies, enrolling a total of 2185 participants (1775 receiving palbociclib, 376 receiving ribociclib, and 34 receiving abemaciclib) with HR+/HER2- breast cancer, mostly females (99.9%), recruited from several countries worldwide (Supplementary Figure S1), and with a mean age ranging between 25 and 92 years. Race was not reported in any of the studies included in the exam. Of them, 763 received concomitant PPI (34.9%). The characteristics of included survival outcome studies are detailed in Table 2.

**Table 2:** Survival outcome studies characteristics and results

Study, year	Study design	Sex (%)	Age, mean (SD/range)	Treatment arm (n)	ECOG (n)	Endocrine sensitive (n)	Menopause (n)	Metastatic sites (n)	Reported PPI (n) ^b^	PFS, HR (95%CI)	OS, HR (95%CI)
Çağlayan, 2023 [30]	OR	F (100)	55.5 (12.8)	Palb (21)	NA	16	NA	NA	Es; La; Om; Pa (NA)^a^	2.54 (1.14–5.64)	NA
				Ribo (20)							
				Palb + PPI (29)	NA	20	NA	NA			
				Ribo + PPI (16)							
Del Re, 2021 [33]	OR	NA	63 (35–86)	Palb (56)	ECOG 0–1 (55)	35	45	V (31)	Es (1); La (42); Om (11); Pan (2)^a^	2.77 (1.62–4.75)	NA
					ECOG 2 (1)			NV (25)			
				Palb + PPI (56)	ECOG 0–1 (54)	36	48	V (24)			
					ECOG 2 (2)			NV (32)			
Del Re, 2022 [32]	OR	NA	59.1 (9.7)	Ribo (78)	ECOG 0–1 (73)	62	60	V (41)	Es (3); La (34); Om (6); Pa (7)^a^	1.17 (0.65–2.14)	NA
					ECOG 2 (5)			NV (26)			
				Ribo + PPI (50)	ECOG 0–1 (47)	44	37	V (36)			
					ECOG 2 (3)			NV (24)			
Eser, 2022a (palbociclib) [31]	OR^a^	NA	59 (32–8)	Palb (40)	ECOG 0–1 (85)^a^	57^a^	55^a^	V (46)^a^	Es (26); La (12); Om (15); Pa (46); Ra (27)^a^	7.85 (2.67–23.05)	NA
				Palb + PPI (65)	ECOG 2 (6)^a^			NV (45)^a^			
Eser, 2022b (ribociclib) [31]	OR^a^	NA	53 (32–87)	Ribo (51)	ECOG 0–1 (106)^a^	58^a^	87^a^	V (71)^a^	Es (26); La (12); Om (15); Pa (46); Ra (27)^a^	2.90 (1.38–6.40)	NA
				Ribo + PPI (61)	ECOG 2 (20)^a^			NV (55)^a^			
Lee, 2023 [27]	OR^a^	F (100)^a^	NA	Palb (966)	NA	819^a^	951^a^	V (253)^a^	De; Es; Il; La; Om; Pa; Ra (NA)^a^	1.76 (1.46–2.13)	2.71 (2.07–3.53)
				Palb + PPI (344)				NV (264)^a^			
Odabas, 2023a (palbociclib) [28]	OR^a^	F (99.0)^a^	58 (25–92)	Palb (63)	ECOG 0–1 (122)^a^	47^a^	92^a^	V (63)^a^	Es (20); La (31); Om (6); Pa (21); Ra (8)^a^	0.98 (0.59–1.65)	NA
				Palb + PPI (57)	ECOG 2–3 (12)^a^			NV (71)^a^			
Odabas, 2023b (ribociclib) [28]	OR^a^	F (99.0)^a^	56 (31–84)	Ribo (71)	ECOG 0–1 (81)^a^	39^a^	73^a^	V (45)^a^	Es (20); La (31); Om (6); Pa (21); Ra (8)^a^	1.71 (0.78–3.78)	NA
				Ribo + PPI (29)	ECOG 2–3 (6)^a^			NV (41)^a^			
Takahashi, 2024a (palbociclib) [26]	OR^a^	F (99.0)^a^	70 (62–75)	Palb (35)	OG 0–1 (53)^a^	NA	52^a^	V (36)^a^	Es (10); La (25); Om (3); Ra (8); Vo (10)^a^	0.94 (0.61–1.46)	1.47 (0.82–2.62)
				Palb + PPI (43)	ECOG 2 (3)^a^			NV (20)^a^			
Takahashi, 2024b (abemaciclib) [26]	OR^a^	F (99.0)^a^	71 (60–75)	Abem (21)	OG 0–1 (52)^a^	NA	48^a^	V (34)^a^	Es (10); La (25); Om (3); Ra (8); Vo (10)^a^	1.3 (0.53–3.17)	1.22 (0.33–4.47)
				Abem + PPI (13)	ECOG 2 (4)^a^			NV (22)^a^			

*Abbreviations: 95%CI* 95% confidence interval, *Abem* abemaciclib, *CDKI* cycline-dependent kinase inhibitors, *De* dexlansoprazole, *ECOG* Eastern Cooperative Oncology Group performance score, *Es* esomeprazole, *F* female, *Il* ilaprazole, *HR* hazard ratio, *La* lansoprazole, *NA* not available, *NV* non-visceral, *O* other, *Om* omeprazole, *OR* observational retrospective, *OS* overall survival, *Palb* palbociclib, *Pa* pantoprazole, *PFS* progression-free survival, *PPI* proton pump inhibitor, *Ra* rabeprazole, *Ribo* ribociclib, *SD* standard deviation, *V* visceral, *Vo* vonoprazan

^a^Values reported for the overall study population

^b^Reported PPI are to be considered for the CDKI + PPI treatment arm only

### Risk of bias assessment for PK outcomes

**Fig. 2:**
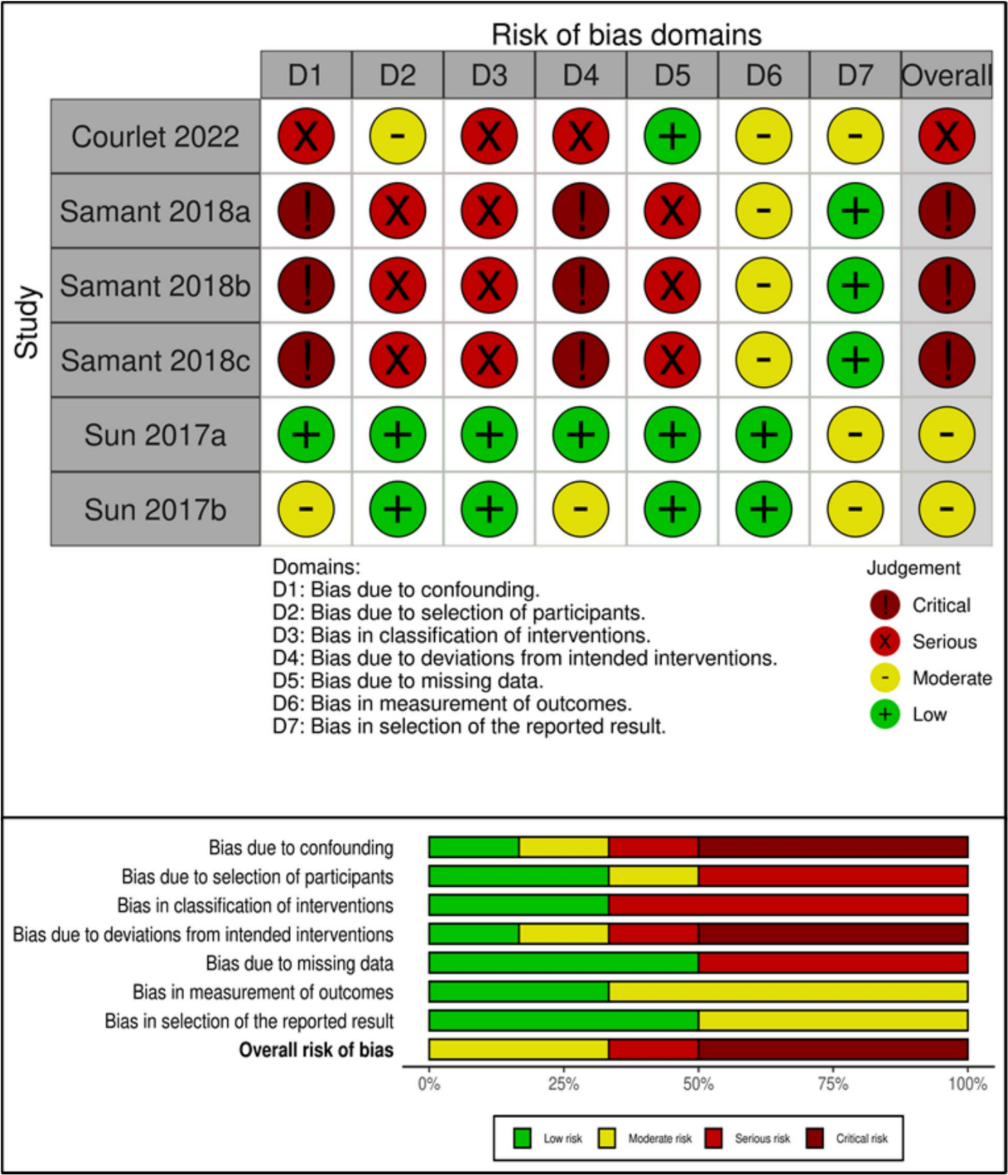
Risk of bias assessment for PK outcome studies

We assessed the ROBINS-I for all six studies reported by the three included papers. Overall, one paper was considered at serious risk of bias due to confounding for lacking information about baseline variables, unclear definitions in the process of participant selection and classification of the intended intervention, and lack of information about cointerventions (bias due to deviation from the intended intervention). A second paper, reporting data from three different studies, was considered a critical risk of bias. All three studies had a critical risk of bias due to confounding and deviations from intended interventions, since baseline variables and cointerventions were not reported; additionally, they were considered at serious risk of bias due to selection of participants, classification of interventions, and missing data since the sample size varied on the basis of different reported outcomes and lacked data about selection and inclusion processes. The last paper, reporting data from two different studies was considered to have an overall moderate risk of bias, and was thus sound for a nonrandomized study. In contrast to the other included studies, we judged the bias in the measurement of the outcomes to be low since the data were collected in the context of prospective clinical trials. The risk of bias assessment for the PK outcome studies is detailed in Fig. 2.

### Risk of bias assessment for survival outcomes

**Fig. 3:**
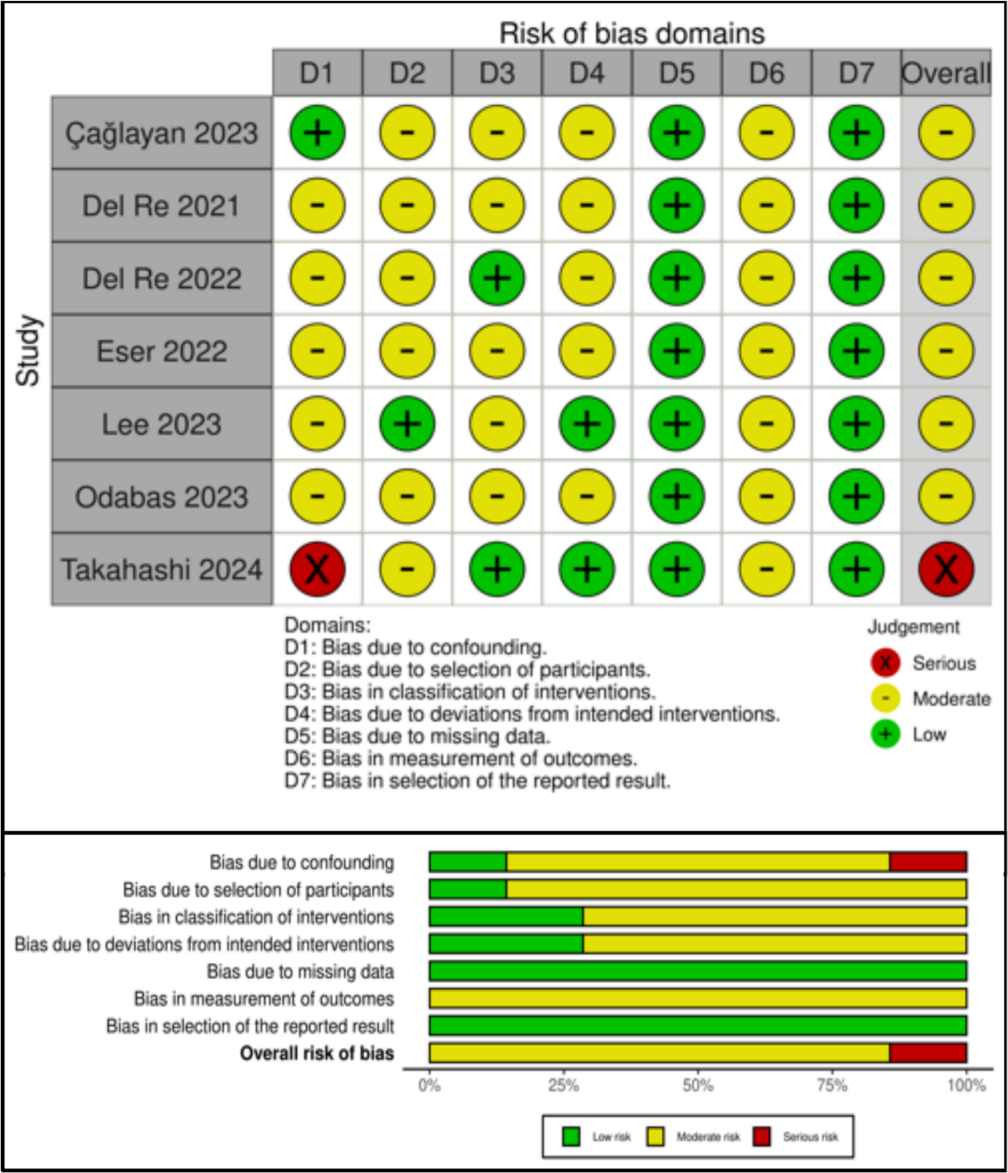
Risk of bias for survival outcome studies

We considered almost all included studies at moderate risk of bias due to confounding since they did not control for Ki-67 except for one study, which we considered at low risk of bias for confounding [30]. One study was considered at serious risk of bias due to confounding since baseline-adjusted results were not reported [32]. Six studies were at moderate risk of bias in the selection of participants since they were unclear or lacked information on the timing of the follow-up and intervention start date. Five studies with limited information on the source of data, particularly if the information used to define intervention groups was recorded at the start of the intervention, were considered at moderate risk of bias for the classification of interventions. We considered five studies at moderate risk of bias due to deviations from the intended intervention since we did not find any reference to verification of adherence to the intervention. All studies were considered at moderate risk of bias in the measurement of outcomes domain because of their retrospective nature, since investigators’ knowledge of the outcome in included patients was likely. All studies were considered at low risk of bias due to missing data and selection of the reported results. The risk of bias assessment for the clinical outcome studies is detailed in Fig. 3.

### Pharmacokinetics outcomes

**Fig. 4:**
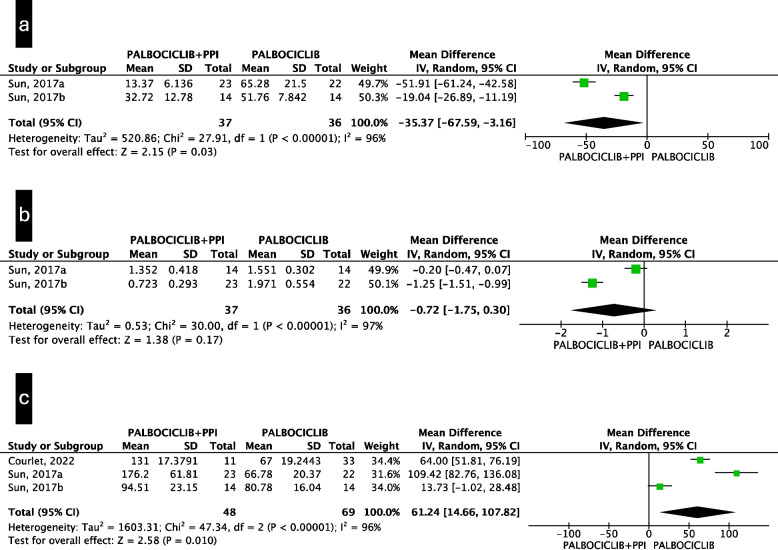
Mean difference (“palbociclib + PPI”- “palbociclib”) meta-analysis for pharmacokinetic outcomes of palbociclib: **a** palbociclib, the mean difference of the arithmetic mean Cmax [ng/mL]; **b** palbociclib, the mean difference of the arithmetic mean AUC [mgh/mL]; **c** palbociclib, mean difference of the arithmetic mean CL/F [L/h]

**Fig. 5:**
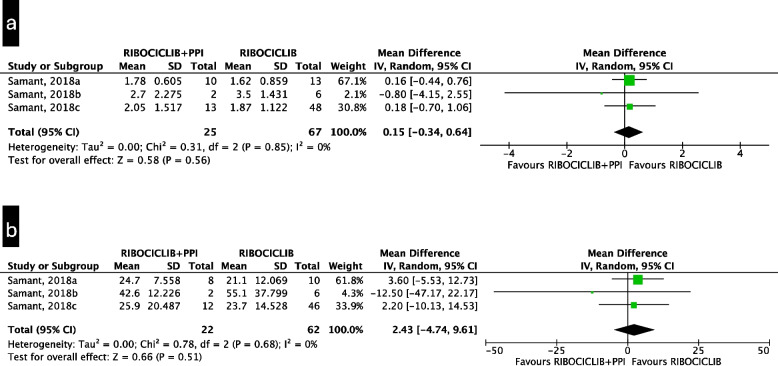
Mean difference (“ribociclib + PPI”- “ribociclib”) meta-analysis for pharmacokinetic outcomes of ribociclib: **a** Ribociclib mean difference of the geometric mean Cmax [mg/mL]; **b** Ribociclib mean difference of the geometric mean AUC [mgh/mL]

When coadministered with palbociclib, PPI significantly reduced the Cmax (MD −35.37 ng/mL; 95%CI from −67.59 to −3.16) and resulted in a nonsignificant reduction in the AUC (MD −0.72 mg·h/mL; 95%CI from −1.75 to 0.30), both with a low quality of evidence (Fig. 4a and b). Moreover, PPIs significantly increased the CL/F (MD 61.24 L/h; 95%CI from 14.66 to 107.82) (Fig. 4c). Comparable results were observed when the geometric means for Cmax and AUC were used (Supplementary Figure S2a and S2b), with very low-quality evidence. On the other hand, the Cmax and AUC of ribociclib did not significantly differ among the PPI users (MD 0.15 mg/mL; 95%CI from −0.34 to 0.64 and MD 2.43 mg·h/mL; 95%CI from −4.74 to 9.61, respectively) (Fig. 5a and b), with a low quality of evidence. No PK outcome data for abemaciclib are currently available. Due to the different risk of bias of the studies included in the CL/F pooled estimates, we performed a sensitivity analysis excluding the study with a higher risk of bias, resulting in a similar effect size, however, it was not statistically significant (Supplementary Figure S3). Funnel plots for the evaluation of publication bias for PK outcomes are presented in Supplementary Figure S4. A summary of the findings of the PK outcome studies is presented in Table 3.

**Table 3:** Summary of findings for PK outcomes

Outcomes	Relative effect (95%CI) with PPI added to CDKI	№ of participants (studies)	Certainty of the evidence (GRADE)	Comments
Palbociclib Cmax	MD −35.37 ng/mL	73	⨁⨁◯◯	The evidence suggests PPI added to palbociclib result in a reduction of palbociclib Cmax
	(−67.59 to −3.16)	(2 non-randomised studies)	Low^a,b^	
Palbociclib AUC	MD −0.72 mgh/mL	73	⨁⨁◯◯	The evidence suggests PPI added to palbociclib may reduce palbociclib AUC
	(−1.75 to 0.3)	(2 non-randomised studies)	Low^b,c^	
Palbociclib CL/F	MD 61.24 L/h	117	⨁◯◯◯	PPI added to palbociclib may increase palbociclib CL/F but the evidence is very uncertain
	(14.66 to 107.82)	(3 non-randomised studies)	Very low^d,e,f^	
Ribociclib Cmax	MD 0.15 mg/mL	92	⨁⨁◯◯	The evidence suggests that PPI added to ribociclib result in little to no difference in Cmax
	(−0.34 to 0.64)	(3 non-randomised studies)	Low^g^	
Ribociclib AUC	MD 2.43 mgh/mL	84	⨁⨁◯◯	The evidence suggests that PPI added to ribociclib result in little to no difference in AUC
	(−4.74 to 9.61)	(3 non-randomised studies)	Low^g^	

GRADE Working Group grades of evidence

High certainty: we are very confident that the true effect lies close to that of the estimate of the effect

Moderate certainty: we are moderately confident in the effect estimate: the true effect is likely to be close to the estimate of the effect, but there is a possibility that it is substantially different

Low certainty: our confidence in the effect estimate is limited: the true effect may be substantially different from the estimate of the effect

Very low certainty: we have very little confidence in the effect estimate: the true effect is likely to be substantially different from the estimate of effect

Explanations:

^a^Serious inconsistency (I^2^ = 96%)

^b^Serious imprecision due to small sample size and large confidence interval ranging from limited to considerable clinical impact

^c^Serious inconsistency (I^2^ = 97%)

^d^One study did not report baseline confounding variables between the population and was considered ad serious risk of bias. However, we chose not to downgrade for risk of bias since a sensitivity analysis excluding this study substantially confirmed the estimated results

^e^Serious inconsistency (I^2^ = 96%)

^f^Serious imprecision due to small sample size and large confidence interval

^g^Serious risk of bias due to possible confounding, selection of participants, classification of intervention, deviations from intended interventions, and missing data

*Abbreviations: 95%CI* 95% confidence interval, *AUC* area under curve, *CDKI* cycline-dependent kinase inhibitors, *CL/F* apparent oral clearance, *Cmax* serum maximum concentration, *PPI* proton pump inhibitors

### Survival outcomes

**Fig. 6:**
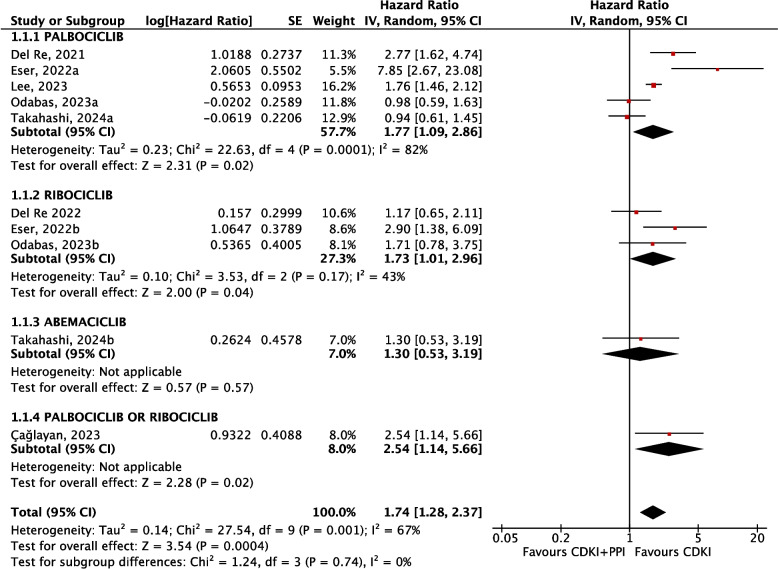
Meta-analysis of PFS outcomes. Hazard of PFS in PPI and CDKI users vs CDKI users

In the overall analysis, the concomitant administration of PPIs with CDKIs resulted in significantly increased hazards for reduced PFS (HR 1.74; 95%CI from 1.28 to 2.37) with moderate quality of evidence (Fig. 6). Consistently, subgroup analyses yielded similar results with concomitant PPIs resulting in significantly increased hazards for reduced PFS both for palbociclib (HR 1.77; 95%CI from 1.09 to 2.86) and ribociclib (HR 1.73; 95%CI from 1.01 to 2.96), with low quality of evidence due to inconsistency and imprecision, respectively. Assessed only in a single study, the concomitant administration of PPIs with abemaciclib resulted in nonsignificant differences in the hazard for PFS (HR 1.30; 95%CI from 0.53 to 3.19), whose quality of evidence was judged very low for very serious imprecision due to severely limited sample size [26].

**Fig. 7:**
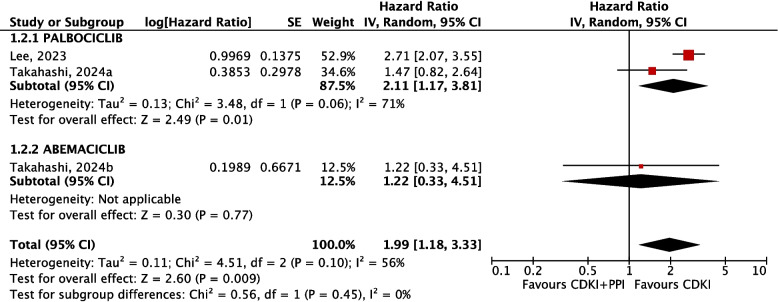
Meta-analysis of OS outcomes. Hazard ratio of OS in PPI and CDKI users vs CDKI users

Similarly, the concomitant administration of PPIs with CDKIs resulted in significantly increased hazards for reduced OS (HR 1.99; 95%CI from 1.18 to 3.33) with a moderate quality of evidence (Fig. 7). This result was mainly based on the effect estimates of the palbociclib subgroup, which resulted in a significantly increased hazard for reduced OS in patients co-administered with PPIs (HR 2.11; 95%CI from 1.17 to 3.81), with a moderate quality of evidence. No data were available for OS evaluation with ribociclib. The single abemaciclib study revealed nonsignificant differences in hazards for OS (HR 1.22; 95%CI from 0.33 to 4.51), with very low quality of evidence due to very serious imprecision. Funnel plots for the evaluation of publication bias for survival outcomes are presented in Supplementary Figure S5. A summary of findings for survival outcomes is presented in Table 4.

**Table 4:** Summary of findings for survival outcomes

Outcomes	Relative effect (95% CI)	№ of participants (studies)	Certainty of the evidence (GRADE)	Comments
PFS Overall	HR 1.72	2185	⨁⨁⨁◯	Concomitant use of PPI with CDKI may reduce PFS
	(1.27 to 2.32)	(7 non-randomized studies)	Moderate^a^	
PFS palbociclib	HR 1.77	1775	⨁⨁◯◯	Concomitant use of PPI with palbociclib may result in PFS reduction
	(1.09 to 2.86)	(5 non-randomized studies)	Low^a,b^	
PFS ribociclib	HR 1.73	376	⨁⨁◯◯	Concomitant use of PPI with ribociclib may result in PFS reduction
	(1.01 to 2.96)	(3 non-randomized studies)	Low^a,c^	
PFS abemaciclib	HR 1.30	34	⨁◯◯◯	The evidence is very uncertain about the effect of concomitant use of PPI in addition to abemaciclib on PFS
	(0.53 to 3.19)	(1 non-randomized study)	Very low^a,d^	
OS Overall	HR 1.99	1422	⨁⨁⨁◯	Concomitant use of PPI with CDKI may reduce OS
	(1.18 to 3.33)	(3 non-randomized studies)	Moderate^a^	
OS palbociclib	HR 2.11	1388	⨁⨁⨁◯	Concomitant use of PPI with palbociclib may result in OS reduction
	(1.17 to 3.81)	(2 non-randomized studies)	Moderate^a,e^	
OS abemaciclib	HR 1.22	34	⨁◯◯◯	The evidence is very uncertain about the effect of concomitant use of PPI in addition to abemaciclib on OS
	(0.33 to 4.51)	(1 non-randomized study)	Very low^a,d^	

GRADE Working Group grades of evidence

High certainty: we are very confident that the true effect lies close to that of the estimate of the effect

Moderate certainty: we are moderately confident in the effect estimate: the true effect is likely to be close to the estimate of the effect, but there is a possibility that it is substantially different

Low certainty: our confidence in the effect estimate is limited: the true effect may be substantially different from the estimate of the effect

Very low certainty: we have very little confidence in the effect estimate: the true effect is likely to be substantially different from the estimate of effect

*Abbreviations: 95%CI *95% confidence interval, *CDKI *cycline-dependent kinase inhibitors, *Cmax *serum maximum concentration, *HR *hazard ratio, *OS *overall survival, *PFS *progression-free survival, *PPI *proton pump inhibitors

^a^We considered the overall ROBINS-I serious; the judgement was mainly due to the absence of adjustment for Ki-67 in the majority of included studies (i.e., bias due to confounding). However, we did not consider this relevant enough to downgrade for risk of bias

^b^Serious inconsistency (I^2^ = 81%), with some studies reporting no substantial effect and others reporting large effect

^c^Serious imprecision due to limited sample size

^d^Very serious imprecision due to severely limited sample size and 95%CI ranging from substantial benefit to substantial harm

^e^Inconsistency was moderately high for this comparison (i2 = 71%); however, since the direction of the effect was similar in included studies, our judgement was to not downgrade for inconsistency

To explain the heterogeneity observed among the studies, we attempted to pool univariate analyses of the included studies for age, ECOG score, metastatic site, endocrine sensitivity, and menopausal status, when available. Unfortunately, these findings did not show any relevant results that could explain this phenomenon (Supplementary Figure S6).

One retrospective study enrolling 82 patients receiving palbociclib, of whom 32 concomitantly received PPIs, did not provide usable results for our quantitative analysis [29]. For this reason, we decided to narratively discuss its results. The study did not show significant differences in median PFS between groups (median 20.6 months; 95%CI from 16.07 to not estimable for the no PPI use arm and median 21.0 months; 95%CI from 15.15 to not estimable for the PPI use arm, *p* = 0.95). The median OS was not reached and thus was not analyzed.

## Discussion and conclusions

Acidic gastric pH is a fundamental condition for the absorption of weak base, such as palbociclib, ribociclib, and abemaciclib. Del Re et al. reported that palbociclib and ribociclib, as expected for weak bases, possess pH-dependent solubilities. Palbociclib solubility decreases from 0.5 mg/mL to 0.05 mg/mL as the pH increases from 3 to 5 [37]. Ribociclib solubility is influenced by pH changes from 2.4 mg/mL when the pH is between 5 and 6 to 0.3–0.8 mg/mL when the pH is ≥ 6.8 [32]. Abemaciclib showed a pH-dependent solubility ranging from 5 mg/mL at pH 6 to 1.58 mg/mL at pH 6.8. Notably, these pH changes are expected to occur with the use of PPIs [41]. Because the decreased gastric solubility may be responsible for the reduced drug absorption, the limited influence of pH on ribociclib may explain the nonsignificant effect on tha Cmax and AUC of the combination of PPIs with ribociclib, at the same time, the sensitivity of palbociclib to pH changes could explain our meta-analysis results indicating that the concomitant use of PPIs reduces palbociclib Cmax and AUC mean values.

We also observed a significant increase in HR for both PFS and OS in the case of co-assumption of CDKI and PPI. PFS and OS are reduced in patients who receive concomitant PPIs with palbociclib confirming the pharmacokinetic data underlying the reduced absorption of CDKIs. In contrast, in our meta-analysis, the subgroup analysis of ribociclib produced pooled estimates for PFS similar to those of the palbociclib subgroup, even if the pharmacokinetic analysis did not demonstrate a significant modulation of ribociclib absorption. However, the sample sizes of studies investigating survival outcomes with ribociclib were remarkably inferior to those of palbociclib, thus reducing the certainty of the evidence due to imprecision. Other authors have shown that the therapeutic index of ribociclib is broader than that of palbociclib, making its efficacy less influenced by pH fluctuations [42]. However, the discrepancy in survival outcomes without significant PK variations remains unresolved. Nevertheless, since PK outcomes and survival outcomes were assessed in different patients and studies, conducting studies investigating both outcomes in the same patients could improve the mechanistic interpretation of the effects of PK on clinical efficacy and limit bias due to confounding factors.

Limited data are available for other processes such as the distribution or metabolism of these molecules, which could explain the interaction between PPIs and CDKIs. CDKIs are metabolized mainly by CYP3A4 with palbociclib and ribociclib being weak and moderate-to-strong inhibitors of this enzyme, respectively. CYP3A4 is partially inhibited by some PPIs such as lansoprazole and rabeprazole [43, 44]. Unfortunately, the limited amount of data for specific PPIs prevented us from performing subgroup analyses of different PPIs to estimate any metabolic interactions. The formation of a metabolite with grater activity than the parent drug could, for example, explain what was previously reported for ribociclib. An interesting study revealed that ribociclib possesses several functional sites that can be targeted by several cytochromes [45]. Recently, James et al. reported that this molecule is metabolized primarily by CYP3A4 (54%) and flavin-containing monooxygenase 3 (FMO3, 36%) producing LEQ803 and CCI283 as the main metabolites, respectively [46]. However, data on files by Novartis suggest that these and other minoritarian metabolites are clinically irrelevant even if they are pharmacologically active [47]. Therefore, the decrease in the efficacy of ribociclib in combination with PPIs cannot be explained by metabolic interference.

The excretion of CDKIs varies between molecules, being mainly renal for palbociclib and ribociclib and biliary for abemaciclib. Data for CL/F are available only for palbociclib [37, 40]. PPI co-administration significantly increased palbociclib clearance and elimination. However, CL/F is the ratio between the clearance value (CL) and the absorbed fraction or bioavailability (F). Since an AUC reduction in PPI users implies a reduction in F, the observed effect on CL/F seems to be related mainly to a decrease in F rather than a real increase in clearance. These findings support the idea that elimination is less involved in the mechanism by which PPIs interact with the bioavailability and efficacy of CDKIs. Given that ribociclib has chemical properties similar to those of palbociclib, it is reasonable to expect a similar behavior for excretion. However, considering the different mechanisms of elimination, it is difficult to imagine any clinically relevant effects of PPIs on abemaciclib excretion, and further PK and clinical studies are pivotal for a better understanding.

Our meta-analysis has several inevitable limitations. First, the outcomes themselves are limited since the reported outcomes are only PFS and, in few cases, OS. The evaluation of other outcomes, such as disease stability or complete/partial response, would provide better insights into the relationship between PPIs and CDKIs. A second weak point is represented by currently available clinical data since they come from observational retrospective studies. These surely allow us to observe the effects in a real-world context, but they are also impacted by uncontrolled confounding variables that only clinical trials, or at least very sound, prospective observational studies can avoid (e.g., fed-state, ethnicity, drug formulation, administered PPI, Ki-67, concomitant medications leading to possible drug‒drug interactions with CDKIs). Finally, the sample size of individual studies is highly variable among different CDKIs. This led to better clinical and pharmacokinetic definitions of the effects of PPIs on palbociclib, followed by ribociclib and abemaciclib. The limited number of patients, especially those treated with abemaciclib, does not allow us to clearly state any clinical involvement of the PPI interaction in the PK data.

We carried out two parallel meta-analyses exploring both PK and clinical outcomes related to co-administration of PPIs with CDKIs. Our results are based only on published scientific articles with exploitable results. We pointed out how PPIs are able to reduce CDKI absorption as well as decrease their clinical efficacy. However, while this effect is clearn for palbociclib, some doubts remain for ribociclib, and even more for abemaciclib. The co-administration of PPIs with abemaciclib is currently being studied in only one study and can thus be considered almost unexplored. Prospective studies are strongly advised to clarify whether the negative interaction is class- or molecule-related. These data suggest that PPI and CDKI co-administration should be avoided unless it is strictly indicated. In these cases, ribociclib or abemaciclib seem to be better options than palbociclib, even though closer monitoring is strongly advised.

## Supplementary Information

Supplementary Material 1.

## Data Availability

Not applicable.

## Abbreviations

CDKI: Cyclin-dependent kinase 4/6 inhibitor
CDKIs: Cyclin-dependent kinase 4/6 inhibitors
HR+: Receptor-positive
HER2-: Receptor 2 negative
PPIs: Proton pump inhibitors
PFS: Progression-free survival
OS: Overall survival
CL/F: Increased the clearance
PRISMA: Reporting Items for Systematic Reviews and Meta-analysis
Cmax: Serum maximum concentration
SD: Standard deviation
CV%: Percentual coefficient of variation
95%CI: 95% Confidence interval
HR: Hazard ratios
ROBINS-I: The Cochrane Risk of Bias in Non-randomized Studies of Interventions
MD: Mean differences
CL: Clearance value
PK: Pharmacokinetics
