# Supplementary material for: Pharmacokinetic interactions and clinical implications of PPIs and CDKIs in breast cancer: a systematic review and meta-analysis
Source: Syst Rev. 2025 Dec 30;15:36. doi: 10.1186/s13643-025-03046-0 (PMC12859863; doi:10.1186/s13643-025-03046-0)
Supplement: Supplementary file 1 — Supplementary Material 1. [file 13643_2025_3046_MOESM1_ESM.docx]

**Additional Information For**

**Pharmacokinetic Interactions and Clinical Implications of PPIs and CDKIs in Breast Cancer: A Systematic Review and Meta-Analysis**

**Appendices**

| **Page 2** | **Supplementary Table S1** – PRISMA- checklist |
| --- | --- |
| **Page 5.** | **Supplementary Table S2** - Search Strategy |
| **Page 8.** | **Supplementary Figure S1.** Geographical distribution of CDKi-PPI studies |
| **Page 9.** | **Supplementary Figure S2.** Meta-analysis of PK outcomes using geometric mean for Palbociclib |
| **Page 9.** | **Supplementary Figure S3.** CL/F sensitivity analysis |
| **Page 10.** | **Supplementary Figure S4.** Funnel plots for pharmacokinetic outcomes |
| **Page 10.** | **Supplementary Figure S5.** Funnel plots for survival outcomes |
| **Page 11.** | **Supplementary Figure S6.** Additional PFS analyses |

**Supplementary Table S1** – PRISMA- checklist

| **Section and Topic** | **Item #** | **Checklist item** | | **Location where item is reported** | | |
| --- | --- | --- | --- | --- | --- | --- |
| **TITLE** | | |  | | |  |
| Title | 1 | Identify the report as a systematic review. | | 1 | | |
| **ABSTRACT** | | |  | | |  |
| Abstract | 2 | See the PRISMA 2020 for Abstracts checklist. | | 2 | | |
| **INTRODUCTION** | | |  | | |  |
| Rationale | 3 | Describe the rationale for the review in the context of existing knowledge. | | 3 | | |
| Objectives | 4 | Provide an explicit statement of the objective(s) or question(s) the review addresses. | | 3 | | |
| **METHODS** | | |  | | |  |
| Eligibility criteria | 5 | Specify the inclusion and exclusion criteria for the review and how studies were grouped for the syntheses. | | 3 | | |
| Information sources | 6 | Specify all databases, registers, websites, organisations, reference lists and other sources searched or consulted to identify studies. Specify the date when each source was last searched or consulted. | | 3 | | |
| Search strategy | 7 | Present the full search strategies for all databases, registers and websites, including any filters and limits used. | | Table S1 | | |
| Selection process | 8 | Specify the methods used to decide whether a study met the inclusion criteria of the review, including how many reviewers screened each record and each report retrieved, whether they worked independently, and if applicable, details of automation tools used in the process. | | 4 | | |
| Data collection process | 9 | Specify the methods used to collect data from reports, including how many reviewers collected data from each report, whether they worked independently, any processes for obtaining or confirming data from study investigators, and if applicable, details of automation tools used in the process. | | 3 | | |
| Data items | 10a | List and define all outcomes for which data were sought. Specify whether all results that were compatible with each outcome domain in each study were sought (e.g. for all measures, time points, analyses), and if not, the methods used to decide which results to collect. | | 4 | | |
|  | 10b | List and define all other variables for which data were sought (e.g. participant and intervention characteristics, funding sources). Describe any assumptions made about any missing or unclear information. | | 4 | | |
| Study risk of bias assessment | 11 | Specify the methods used to assess risk of bias in the included studies, including details of the tool(s) used, how many reviewers assessed each study and whether they worked independently, and if applicable, details of automation tools used in the process. | | 4 | | |
| Effect measures | 12 | Specify for each outcome the effect measure(s) (e.g. risk ratio, mean difference) used in the synthesis or presentation of results. | | 4 | | |
| Synthesis methods | 13a | Describe the processes used to decide which studies were eligible for each synthesis (e.g. tabulating the study intervention characteristics and comparing against the planned groups for each synthesis (item #5)). | | 4 | | |
|  | 13b | Describe any methods required to prepare the data for presentation or synthesis, such as handling of missing summary statistics, or data conversions. | | 4 | | |
|  | 13c | Describe any methods used to tabulate or visually display results of individual studies and syntheses. | | Table 1, Table 2, table 3, Table 4 | | |
|  | 13d | Describe any methods used to synthesize results and provide a rationale for the choice(s). If meta-analysis was performed, describe the model(s), method(s) to identify the presence and extent of statistical heterogeneity, and software package(s) used. | | 4 | | |
|  | 13e | Describe any methods used to explore possible causes of heterogeneity among study results (e.g. subgroup analysis, meta-regression). | | 6,7 | | |
|  | 13f | Describe any sensitivity analyses conducted to assess robustness of the synthesized results. | | S2,S3,S6 | | |
| Reporting bias assessment | 14 | Describe any methods used to assess risk of bias due to missing results in a synthesis (arising from reporting biases). | | 4 | | |
| Certainty assessment | 15 | Describe any methods used to assess certainty (or confidence) in the body of evidence for an outcome. | | 4 | | |
| **RESULTS** | | |  | |  |  |
| Study selection | 16a | Describe the results of the search and selection process, from the number of records identified in the search to the number of studies included in the review, ideally using a flow diagram. | | 5 | | |
|  | 16b | Cite studies that might appear to meet the inclusion criteria, but which were excluded, and explain why they were excluded. | | 5 | | |
| Study characteristics | 17 | Cite each included study and present its characteristics. | | Table 1 and Table2 | | |
| Risk of bias in studies | 18 | Present assessments of risk of bias for each included study. | | Fig 2 and Fig3 | | |
| Results of individual studies | 19 | For all outcomes, present, for each study: (a) summary statistics for each group (where appropriate) and (b) an effect estimate and its precision (e.g. confidence/credible interval), ideally using structured tables or plots. | | Fig 4,5,6,7 | | |
| Results of syntheses | 20a | For each synthesis, briefly summarise the characteristics and risk of bias among contributing studies. | | Fig2 and Fig3 | | |
|  | 20b | Present results of all statistical syntheses conducted. If meta-analysis was done, present for each the summary estimate and its precision (e.g. confidence/credible interval) and measures of statistical heterogeneity. If comparing groups, describe the direction of the effect. | | Fig. 4,5,6, and 7 | | |
|  | 20c | Present results of all investigations of possible causes of heterogeneity among study results. | | Fig. S4, S5 and S6 | | |
|  | 20d | Present results of all sensitivity analyses conducted to assess the robustness of the synthesized results. | | Fig. 4, 5, 6, 7 and S6 | | |
| Reporting biases | 21 | Present assessments of risk of bias due to missing results (arising from reporting biases) for each synthesis assessed. | | Fig 2 and Fig 3 | | |
| Certainty of evidence | 22 | Present assessments of certainty (or confidence) in the body of evidence for each outcome assessed. | | Table 3 and Table 4 | | |
| **DISCUSSION** | | |  | | |  |
| Discussion | 23a | Provide a general interpretation of the results in the context of other evidence. | | 7,8 | | |
|  | 23b | Discuss any limitations of the evidence included in the review. | | 8 | | |
|  | 23c | Discuss any limitations of the review processes used. | | 8 | | |
|  | 23d | Discuss implications of the results for practice, policy, and future research. | | 9 | | |
| **OTHER INFORMATION** | | |  | | |  |
| Registration and protocol | 24a | Provide registration information for the review, including register name and registration number, or state that the review was not registered. | | 1,3 | | |
|  | 24b | Indicate where the review protocol can be accessed, or state that a protocol was not prepared. | | 1,3 | | |
|  | 24c | Describe and explain any amendments to information provided at registration or in the protocol. | | 3 | | |
| Support | 25 | Describe sources of financial or non-financial support for the review, and the role of the funders or sponsors in the review. | | 14 | | |
| Competing interests | 26 | Declare any competing interests of review authors. | | 14 | | |
| Availability of data, code and other materials | 27 | Report which of the following are publicly available and where they can be found: template data collection forms; data extracted from included studies; data used for all analyses; analytic code; any other materials used in the review. | | - | | |

**Supplementary Table S2 - Search Strategy**

| **Search Strategy Web of Science** |
| --- |
| AB=(cdki) OR AB=(cyclin dependent kinase inhibitor) OR AB=(palbociclib) OR AB=(ibrance) OR AB=(pd 0332991) OR AB=(ribociclib) OR AB=(kisqali) OR AB=(lee011) OR AB=(abemaciclib) OR AB=(verzenio) OR AB=(ly2385219) OR TS=(cdki) OR TS=(cyclin dependent kinase inhibitor) OR TS=(palbociclib) OR TS=(ibrance) OR TS=(pd 0332991) OR TS=(ribociclib) OR TS=(kisqali) OR TS=(lee011) OR TS=(abemaciclib) OR TS=(verzenio) OR TS=(ly2385219) AND AB=(Pantoprazole) OR AB=(Rabeprazole) OR AB=(Esomeprazole) OR AB=(Omeprazole) OR AB=(Dexlansoprazole) OR AB=(Lansoprazole) OR AB=(2-Pyridinylmethylsulfinylbenzimidazoles) OR AB=(Proton Pump Inhibitors) OR AB=(Pantoprazole) OR AB=(Rabeprazole) OR AB=(Esomeprazole) OR AB=(Omeprazole) OR AB=(Dexlansoprazole) OR AB=(Lansoprazole) OR AB=(2-Pyridinylmethylsulfinylbenzimidazoles) OR AB=(Proton Pump Inhibitors) OR TS=(Pantoprazole) OR TS=(Rabeprazole) OR TS=(Esomeprazole) OR TS=(Omeprazole) OR TS=(Dexlansoprazole) OR TS=(Lansoprazole) OR TS=(2-Pyridinylmethylsulfinylbenzimidazoles) OR TS=(Proton Pump Inhibitors) OR TS=(Pantoprazole) OR TS=(Rabeprazole) OR TS=(Esomeprazole) OR TS=(Omeprazole) OR TS=(Dexlansoprazole) OR TS=(Lansoprazole) OR TS=(2-Pyridinylmethylsulfinylbenzimidazoles) OR TS=(Proton Pump Inhibitors) |
| Results: 31 papers |

| **Search Strategy Cochrane Library** |
| --- |
| **#**1 MeSH descriptor: [Cyclin-Dependent Kinase Inhibitor Proteins] explode all trees 128 |
| #2 (palbociclib):ti,ab,kw 638 |
| #3 (cdki):ti,ab,kw 14 |
| #4 (ibrance):ti,ab,kw 28 |
| #5 (pd 0332991):ti,ab,kw 48 |
| #6 (ribociclib):ti,ab,kw 342 |
| #7 (KISQALI):ti,ab,kw 11 |
| #8 (lee011):ti,ab,kw 53 |
| #9 (abemaciclib):ti,ab,kw 345 |
| #10 (ly2385219):ti,ab,kw 0 |
| #11 (verzenio):ti,ab,kw 3 |
| #12 (cyclin dependent kinase inhibitor):ti,ab,kw 433 |
| **#13 #1 OR #2 OR #3 OR #4 OR #5 OR #6 OR #7 OR #8 OR #9 OR #10 OR #11 OR #12** **1463** |
| #14 ("pantoprazole"):ti,ab,kw 1423 |
| #15 (Rabeprazole):ti,ab,kw 1173 |
| #16 (Esomeprazole):ti,ab,kw 1831 |
| #17 (Omeprazole):ti,ab,kw 4731 |
| #18 (Dexlansoprazole):ti,ab,kw 285 |
| #19 (Lansoprazole):ti,ab,kw 1670 |
| #20 MeSH descriptor: [Proton Pump Inhibitors] explode all trees 1973 |
| #21 MeSH descriptor: [Pantoprazole] explode all trees 620 |
| #22 MeSH descriptor: [Esomeprazole] explode all trees 915 |
| #23 MeSH descriptor: [Rabeprazole] explode all trees 573 |
| #24 MeSH descriptor: [Omeprazole] explode all trees 3526 |
| #25 MeSH descriptor: [Dexlansoprazole] explode all trees 217 |
| #26 MeSH descriptor: [Lansoprazole] explode all trees 919 |
| #27 (Proton Pump Inhibitors):ti,ab,kw 3358 |
| **#28 #14 or #15 or #16 or #17 or #18 or #19 or #20 or #21 or #22 or #23 or #24 or #25 or #25 or #26 or #27 10195** |
| **#29 #13 and #28 8** |
| Results: 8 papers |

| **Search Strategy Pubmed** |
| --- |
| ((("cdki"[Title/Abstract]) OR ("cyclin dependent kinase inhibitor"[Title/Abstract])) OR (((((("palbociclib" [Supplementary Concept]) OR ("palbociclib"[Title/Abstract])) OR ("ibrance"[Title/Abstract])) OR ("pd 0332991"[Title/Abstract])) OR (((("ribociclib" [Supplementary Concept]) OR ("ribociclib"[Title/Abstract])) OR ("kisqali"[Title/Abstract])) OR ("lee011"[Title/Abstract]))) OR ((((("abemaciclib" [Supplementary Concept]) OR ("abemaciclib"[Title/Abstract])) OR ("verzenio"[Title/Abstract])) OR ("ly2385219"[Title/Abstract]))))) AND (("Pantoprazole"[Mesh]) OR ("Rabeprazole"[Mesh]) OR ("Esomeprazole"[Mesh]) OR ("Omeprazole"[Mesh]) OR ("Dexlansoprazole"[Mesh]) OR ("Lansoprazole"[Mesh]) OR ("2-Pyridinylmethylsulfinylbenzimidazoles"[Mesh]) OR ("Proton Pump Inhibitors"[Mesh]) OR (Pantoprazole[Title/Abstract]) OR (Rabeprazole[Title/Abstract]) OR (Esomeprazole[Title/Abstract]) OR (Omeprazole[Title/Abstract]) OR (Dexlansoprazole[Title/Abstract]) OR (Lansoprazole[Title/Abstract]) OR (2-Pyridinylmethylsulfinylbenzimidazoles[Title/Abstract]) OR (Proton Pump Inhibitors[Title/Abstract])) |
| Results: 25 |

| **Search of strategy Embase** |
| --- |
| #1 'proton pump inhibitor'/exp OR 'gastric proton pump inhibitor' OR 'hydrogen potassium adenosine triphosphatase inhibitor' OR 'hydrogen potassium atpase inhibitor' OR 'proton pump inhibitor' OR 'proton pump inhibitors' OR 'pantoprazole'/exp OR '5 difluoromethoxy 2 [ (3, 4 dimethoxy 2 pyridyl) methylsulfinyl] 1h benzimidazole' OR '6 (difluoromethoxy) 2 [ (3, 4 dimethoxy 2 pyridinyl) methylsulfinyl] 1h benzimidazole' OR '6 (difluoromethoxy) 2 [ (3, 4 dimethoxypyridin 2 yl) methylsulfinyl] 1h benzimidazole' OR 'acidwell' OR 'acipan' OR 'alapanzol' OR 'anagastra' OR 'anesteloc' OR 'apton' OR 'b 8610 023' OR 'b 8610 23' OR 'b8610 023' OR 'b8610 23' OR 'berozol' OR 'brandocare' OR 'branzol' OR 'by 1023' OR 'by1023' OR 'citrel' OR 'contix' OR 'controloc' OR 'controloc control' OR 'dicarbocalm control' OR 'digespan' OR 'dosanloc' OR 'dz 2352a' OR 'dz2352a' OR 'eugastrol reflusso' OR 'eupantol' OR 'gastroloc hexal' OR 'gastrostad' OR 'gaxtron start' OR 'gerdin' OR 'gerdin max' OR 'inipant' OR 'inipepsia' OR 'inipom' OR 'inipomp' OR 'inizol' OR 'ippracid' OR 'ipraalox' OR 'kairol' OR 'maalox control' OR 'maalox reflusso' OR 'nedis' OR 'noacid' OR 'nolpacid' OR 'nolpanta' OR 'nolpaza control' OR 'normogastrol' OR 'ozzion' OR 'pacid' OR 'pangest' OR 'panprazox' OR 'panrazol' OR 'pantacid flux' OR 'pantecta' OR 'pantecta control' OR 'pantip' OR 'pantium' OR 'panto aiwa' OR 'panto aristo' OR 'panto tad' OR 'pantodac' OR 'pantodar' OR 'pantofir' OR 'pantoflux' OR 'pantogastrix' OR 'pantogen' OR 'pantoloc' OR 'pantoloc control' OR 'pantomac' OR 'pantomol' OR 'pantomyl' OR 'pantop' OR 'pantopan' OR 'pantopra-q' OR 'pantopraz' OR 'pantopraz bio' OR 'pantoprazole' OR 'pantoprazole sodium' OR 'pantoprazole sodium sesquihydrate' OR 'pantopro' OR 'pantoprol' OR 'pantorena' OR 'pantorex' OR 'pantosec' OR 'pantozol' OR 'pantozol control' OR 'pantup' OR 'panzilan' OR 'panzol' OR 'panzol pro' OR 'peptazol' OR 'pepticus' OR 'pf 05208751' OR 'pf05208751' OR 'piador' OR 'praz-up' OR 'prazolan (pantoprazole)' OR 'pronzek' OR 'protium' OR 'protizole' OR 'protonix' OR 'protonix iv' OR 'ranloc' OR 'ranloc med' OR 'rifun' OR 'rifun 40' OR 'roxitrol' OR 'rvg 22106' OR 'rvg22106' OR 'sanopaz' OR 'sedipanto' OR 'seltraz' OR 'sk and f 96022' OR 'skf 96022' OR 'skf96022' OR 'sodac' OR 'somac' OR 'somac control' OR 'tecnozol' OR 'tecta' OR 'ulcepraz' OR 'ulceron' OR 'ulcotenal' OR 'ulprix' OR 'way 140951' OR 'way140951' OR 'xoolam reflusso' OR 'zencopan' OR 'zipantola' OR 'zipantola protect' OR 'ziprol' OR 'zolepant' OR 'zolium' OR 'zoltex' OR 'zoprax' OR 'zurcal' OR 'zurcale' OR 'zurcazol' OR 'rabeprazole'/exp OR 'esomeprazole'/exp OR 'omeprazole'/exp OR 'dexlansoprazole'/exp OR 'lansoprazole'/exp OR '2 [[(2 pyridyl)methyl]sulfinyl]benzimidazole derivative'/exp |
| #2 cdki OR 'cyclin dependent kinase inhibitor'/exp OR 'cyclin dependent kinase inhibitor' OR 'cyclin dependent kinase inhibitor protein' OR 'cyclin dependent kinase inhibitor proteins' OR 'cyclin dependent protein kinase inhibitor' OR 'cyclin-dependent kinase inhibitor proteins' OR 'palbociclib'/exp OR '6 acetyl 8 cyclopentyl 5 methyl 2 [ [5 (1 piperazinyl) 2 pyridinyl] amino] pyrido [2, 3 d] pyrimidin 7 (8h) one' OR '6 acetyl 8 cyclopentyl 5 methyl 2 [ [5 (piperazin 1 yl) pyridin 2 yl] amino] pyrido [2, 3 d] pyrimidin 7 (8h) one' OR '6 acetyl 8 cyclopentyl 5 methyl 2 [5 (1 piperazinyl) 2 pyridinylamino] 8h pyrido [2, 3 d] pyrimidin 7 one' OR '6 acetyl 8 cyclopentyl 5 methyl 2 [5 (piperazin 1 yl) pyridin 2 ylamino] 8h pyrido [2, 3 d] pyrimidin 7 one' OR 'ibrance' OR 'palbociclib' OR 'palbociclib isethionate' OR 'pd 0332991' OR 'pd 0332991 0054' OR 'pd 0332991-0054' OR 'pd 332991' OR 'pd0332991' OR 'pd0332991 0054' OR 'pd0332991-0054' OR 'pd332991' OR 'pf 00080665 73' OR 'pf 00080665-73' OR 'pf00080665 73' OR 'pf00080665-73' OR 'ro 4991855' OR 'ro4991855' OR 'ribociclib'/exp OR '7 cyclopentyl n, n dimethyl 2 [ [5 (1 piperazinyl) 2 pyridinyl] amino] 7h pyrrolo [2, 3 d] pyrimidine 6 carboxamide' OR '7 cyclopentyl n, n dimethyl 2 [ [5 (piperazin 1 yl) pyridin 2 yl] amino] 7h pyrrolo [2, 3 d] pyrimidine 6 carboxamide' OR 'kisqali' OR 'lee 011' OR 'lee 011a' OR 'lee 011bba' OR 'lee 11' OR 'lee 11a' OR 'lee 11bba' OR 'lee011' OR 'lee011a' OR 'lee011bba' OR 'lee11' OR 'lee11a' OR 'lee11bba' OR 'ribociclib' OR 'ribociclib butanedioate' OR 'ribociclib succinate' OR 'abemaciclib'/exp OR '[5 (4 ethyl 1 piperazinylmethyl) 2 pyridinyl] [5 fluoro 4 (7 fluoro 3 isopropyl 2 methyl 5 (3h) benzoimidazolyl) 2 pyrimidinyl] amine' OR '[5 (4 ethyl 1 piperazinylmethyl) 2 pyridinyl] [5 fluoro 4 [7 fluoro 3 (1 methylethyl) 2 methyl 5 (3h) benzimidazolyl] 2 pyrimidinyl] amine' OR '[5 (4 ethyl 1 piperazinylmethyl) 2 pyridinyl] [5 fluoro 4 [7 fluoro 3 (1 methylethyl) 2 methyl 5 (3h) benzoimidazolyl] 2 pyrimidinyl] amine' OR '[5 (4 ethyl 1 piperazinylmethyl) 2 pyridinyl] [5 fluoro 4 [7 fluoro 3 (2 propanyl) 2 methyl 5 (3h) benzimidazolyl] 2 pyrimidinyl] amine' OR '[5 (4 ethyl 1 piperazinylmethyl) 2 pyridinyl] [5 fluoro 4 [7 fluoro 3 (2 propanyl) 2 methyl 5 (3h) benzoimidazolyl] 2 pyrimidinyl] amine' OR '[5 (4 ethyl 1 piperazinylmethyl) 2 pyridinyl] [5 fluoro 4 [7 fluoro 3 isopropyl 2 methyl 5 (3h) benzimidazolyl] 2 pyrimidinyl] amine' OR '[5 (4 ethylpiperazin 1 ylmethyl) pyridin 2 yl] [5 fluoro 4 (7 fluoro 3 (1 methylethyl) 2 methyl 3h benzimidazol 5 yl) pyrimidin 2 yl] amine' OR '[5 (4 ethylpiperazin 1 ylmethyl) pyridin 2 yl] [5 fluoro 4 [7 fluoro 3 (1 methylethyl) 2 methyl 3h benzoimidazol 5 yl] pyrimidin 2 yl] amine' OR '[5 (4 ethylpiperazin 1 ylmethyl) pyridin 2 yl] [5 fluoro 4 [7 fluoro 3 (propan 2 yl) 2 methyl 3h benzimidazol 5 yl] pyrimidin 2 yl] amine' OR '[5 (4 ethylpiperazin 1 ylmethyl) pyridin 2 yl] [5 fluoro 4 [7 fluoro 3 (propan 2 yl) 2 methyl 3h benzoimidazol 5 yl] pyrimidin 2 yl] amine' OR '[5 (4 ethylpiperazin 1 ylmethyl) pyridin 2 yl] [5 fluoro 4 [7 fluoro 3 isopropyl 2 methyl 3h benzimidazol 5 yl] pyrimidin 2 yl] amine' OR '[5 (4 ethylpiperazin 1 ylmethyl) pyridin 2 yl] [5 fluoro 4 [7 fluoro 3 isopropyl 2 methyl 3h benzoimidazol 5 yl] pyrimidin 2 yl] amine' OR 'abemaciclib' OR 'abemaciclib mesilate' OR 'abemaciclib mesylate' OR 'abemaciclib methanesulfonate' OR 'bemaciclib' OR 'ly 2835210' OR 'ly 2835219' OR 'ly2835210' OR 'ly2835219' OR 'n [5 [ (4 ethyl 1 piperazinyl) methyl] 2 pyridinyl] 5 fluoro 4 (4 fluoro 1 isopropyl 2 methyl 1h benzimidazol 6 yl) 2 pyrimidinamine' OR 'n [5 [ (4 ethyl 1 piperazinyl) methyl] 2 pyridinyl] 5 fluoro 4 (4 fluoro 1 isopropyl 2 methyl 6 (1h) benzo [d] imidazolyl) 2 pyrimidinamine' OR 'n [5 [ (4 ethyl 1 piperazinyl) methyl] 2 pyridinyl] 5 fluoro 4 [4 fluoro 2 methyl 1 (1 methylethyl) 1h benzimidazol 6 yl] 2 pyrimidinamine' OR 'n [5 [ (4 ethyl 1 piperazinyl) methyl] 2 pyridinyl] 5 fluoro 4 [4 fluoro 2 methyl 1 (2 propanyl) 6 (1h) benzimidazolyl] 2 pyrimidinamine' OR 'n [5 [ (4 ethyl 1 piperazinyl) methyl] 2 pyridinyl] 5 fluoro 4 [4 fluoro 2 methyl 1 isopropyl 1h benzimidazol 6 yl] 2 pyrimidinamine' OR 'n [5 [ (4 ethylpiperazin 1 yl) methyl] 2 pyridinyl] 5 fluoro 4 (4 fluoro 1 isopropyl 2 methyl 1h benzimidazol 6 yl) 2 pyrimidinamine' OR 'n [5 [ (4 ethylpiperazin 1 yl) methyl] pyridin 2 yl] 5 fluoro 4 (4 fluoro 1 isopropyl 2 methyl 1h benzo [d] imidazol 6 yl) pyrimidin 2 amine' OR 'n [5 [ (4 ethylpiperazin 1 yl) methyl] pyridin 2 yl] 5 fluoro 4 [4 fluoro 2 methyl 1 (1 methylethyl) 1h benzimidazol 6 yl] pyrimidin 2 amine' OR 'n [5 [ (4 ethylpiperazin 1 yl) methyl] pyridin 2 yl] 5 fluoro 4 [4 fluoro 2 methyl 1 (propan 2 yl) 1h benzimidazol 6 yl] pyrimidin 2 amine' OR 'verzenio' OR 'verzenios' |
| **#3 #1 AND #2** |
| Results: 187 |


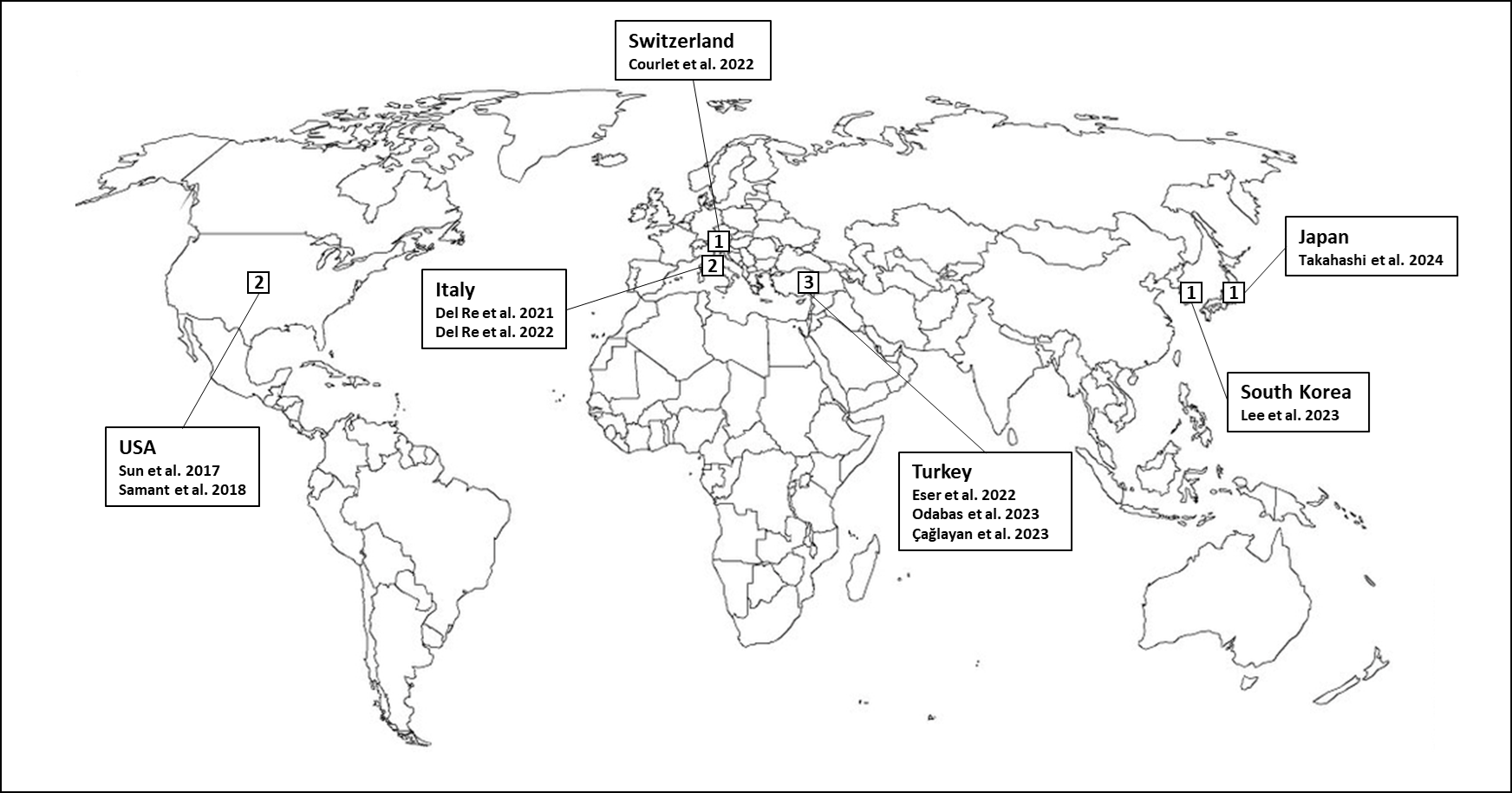


**Supplementary Figure S1** - Geographical distribution of CDKI-PPI studies.

**
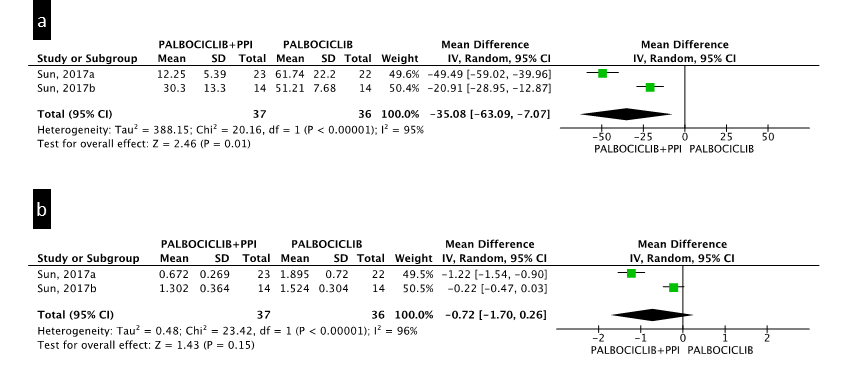
**

**Supplementary Figure S2** - Additional pharmacokinetic analyses. (a)Palbociclib Cmax - geometric mean; (b) Palbociclib AUC - geometric mean.

**
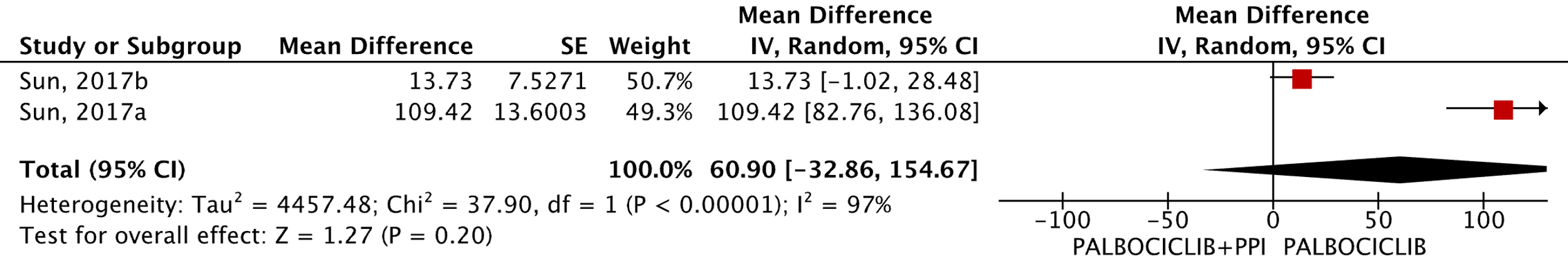
Supplementary Figure S3** - Additional pharmacokinetic analyses for CL/F excluding the study with higher risk of bias.


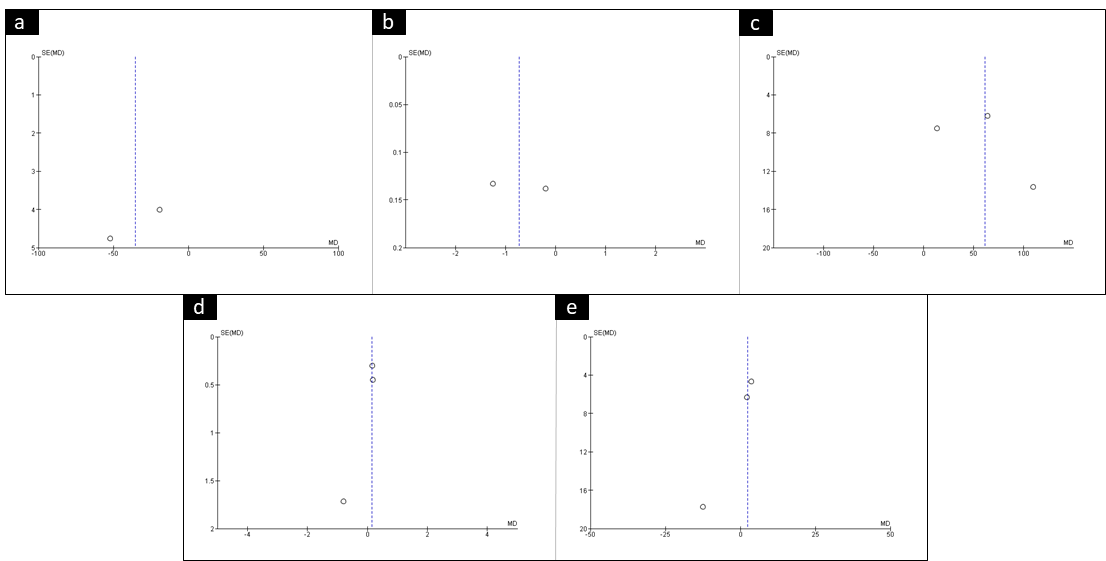


**Supplementary Figure S4** - Funnel plots for pharmacokinetic outcomes. (a)Palbociclib Cmax; (b) Palbociclib AUC; (c) Palbociclib CL/F; (d) Ribociclib Cmax; (e) Ribociclib AUC.


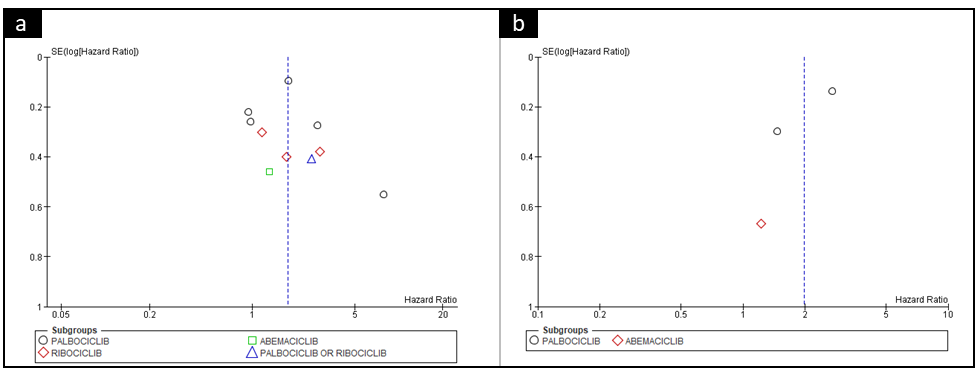


**Supplementary Figure S5** - Funnel plots for clinical outcomes. (a) PFS; (b) OS.


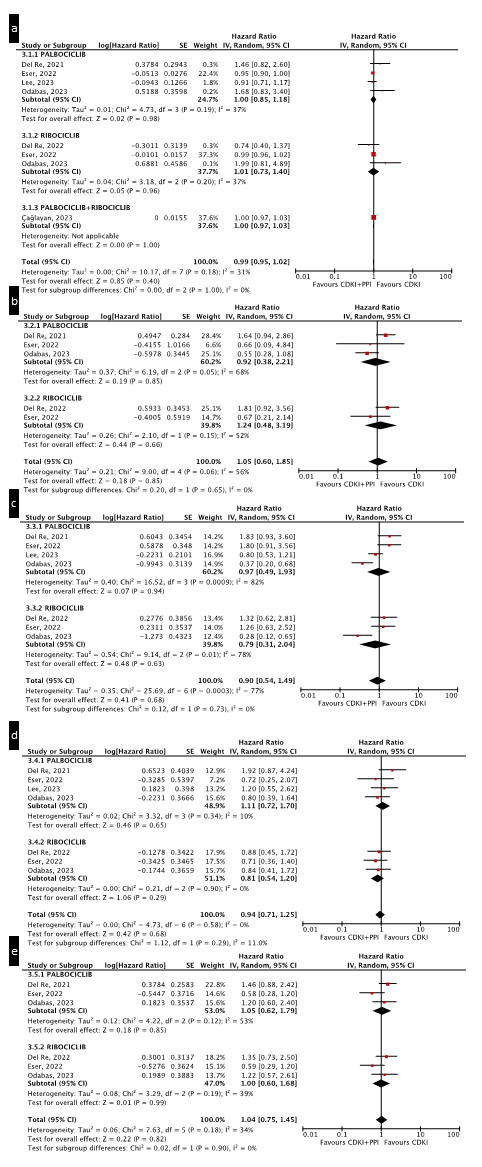


**Supplementary Figure S6** - Additional PFS analyses by (a) Age, (b) ECOG score, (c) Endocrine therapy resistance , (d) Menopausal status, (e) Metastatic sites.
